# Staphylococcal Enterotoxin Genes in Coagulase-Negative Staphylococci—Stability, Expression, and Genomic Context

**DOI:** 10.3390/ijms23052560

**Published:** 2022-02-25

**Authors:** Sylwia Banaszkiewicz, Ewa Wałecka-Zacharska, Justyna Schubert, Aleksandra Tabiś, Jarosław Król, Tadeusz Stefaniak, Ewelina Węsierska, Jacek Bania

**Affiliations:** 1 Department of Food Hygiene and Consumer Health Protection, Wroclaw University of Environmental and Life Sciences, 50-375 Wroclaw, Poland; sylwia.banaszkiewicz@upwr.edu.pl (S.B.); ewa.walecka@upwr.edu.pl (E.W.-Z.); justyna.schubert@gmail.com (J.S.); aleksandra.tabis@upwr.edu.pl (A.T.); 2 Department of Pathology, Division of Microbiology, Wroclaw University of Environmental and Life Sciences, 50-375 Wroclaw, Poland; jaroslaw.krol@upwr.edu.pl; 3 Department of Immunology, Pathophysiology and Veterinary Preventive Medicine, Wroclaw University of Environmental and Life Sciences, 50-375 Wroclaw, Poland; tadeusz.stefaniak@upwr.edu.pl; 4 Department of Animal Product Technology, University of Agriculture in Krakow, 30-239 Krakow, Poland; ewelina.wesierska@urk.edu.pl

**Keywords:** staphylococcal enterotoxins, PCR-detection, expression, *S. aureus*, *S. epidermidis*, coagulase-negative staphylococci (CoNS)

## Abstract

In the current study, we screened a collection of coagulase-negative staphylococci (CoNS) isolates for orthologues of staphylococcal enterotoxins (SEs) involved in *S. aureus*-related staphylococcal food poisoning (SFP). The amplicons corresponding to SEs were detected in *S. chromogenes*, *S. epidermidis*, *S. haemolyticus*, *S. borealis*, *S. pasteuri*, *S. saprophyticus*, *S. vitulinus*, *S. warneri*, and *S. xylosus*. All amplicons were sequenced and identified as parts of known *S. aureus* or *S. epidermidis* SE genes. Quantitative real-time PCR allowed determining the relative copy number of each SE amplicon. A significant portion of the amplicons of the *sea*, *seb*, *sec*, and *seh* genes occurred at low copy numbers. Only the amplicons of the *sec* gene identified in three isolates of *S. epidermidis* displayed relative copy numbers comparable to *sec* in the reference enterotoxigenic *S. aureus* and *S. epidermidis* strains. Consecutive passages in microbiological media of selected CoNS isolates carrying low copy numbers of *sea*, *seb*, *sec*, and *seh* genes resulted in a decrease of gene copy number. *S. epidermidis* isolates harbored a high copy number of *sec*, which remained stable over the passages. We demonstrated that enterotoxin genes may occur at highly variable copy numbers in CoNS. However, we could identify enterotoxin genes only in whole-genome sequences of CoNS carrying them in a stable form at high copy numbers. Only those enterotoxins were expressed at the protein level. Our results indicate that PCR-based detection of enterotoxin genes in CoNS should always require an additional control, like analysis of their presence in the bacterial genome. We also demonstrate *S. epidermidis* as a CoNS species harboring SE genes in a stable form at a specific chromosome site and expressing them as a protein.

## 1. Introduction

Staphylococcal enterotoxins (SEs) produced by *S. aureus* are recognized causes of staphylococcal food poisoning (SFP) [1]. Coagulase-negative staphylococci (CoNS) for a long time were considered only as commensal bacteria, unable to cause infection, and their presence in clinical material was considered as contamination with bacteria colonizing the skin. Nevertheless, numerous studies imply that coagulase-negative staphylococci may also be enterotoxigenic and potentially cause food poisoning. The first descriptions of possible CoNS food poisoning appeared as early as in the 1950s and 1970s [2,3]. The first CoNS strains to produce TSST-1 and SEA enterotoxins were identified in the second half of the 1980s and came from cases of toxic shock in humans [4]. Since then, enterotoxigenic coagulase-negative staphylococci have been identified repeatedly in samples derived from human clinical material, as well as from cases of mastitis in cattle and other ruminants [5,6,7,8].

The number of reports on the enterotoxigenicity of CoNS has been increasing. The method most commonly used for determining the presence of enterotoxin genes is PCR, allowing the detection of small copy numbers of a given gene [9,10,11,12]. However, there are insufficient data about the structure of genetic elements containing enterotoxin genes in the genomes of CoNS and the expression of these genes. Some researchers have observed that the intensity of PCR amplicons corresponding to SEs genes is significantly lower for CoNS than *S. aureus*, despite using comparable amounts of genomic DNA. In addition, a gradual or complete loss of PCR signal, as a result of subsequent passages of bacteria, has been reported [9,13]. Such phenomenon has not been noted for enterotoxigenic *S. aureus* strains. Scientists have hypothesized that staphylococcal enterotoxin genes in some CoNS isolates are unstable and the cell population of such isolates is heterogenic in terms of toxin gene content. As a result of cell division, only some cells may carry genetic elements encoding enterotoxin genes [9].

There are scarce data on the possibility of the acquisition of elements bearing enterotoxin genes by staphylococci. In enterotoxigenic *S. aureus*, the genes encoding staphylococcal enterotoxins are located on mobile genetic elements such as plasmids, prophages, *S. aureus* pathogenicity islands (SaPIs), genomic islands (νSa), or in the proximity of staphylococcal chromosome cassette elements (SCC) [1]. Such locations potentially enable horizontal gene transfer (HGT) between bacteria. To date, HGT between *S. aureus* strains [14,15,16], staphylococcal species (SaPI transfer from *S. aureus* to *S. xylosus*), and between genera of bacteria (SaPI transfer from *S. aureus* to *L. monocytogenes*) [17] has been described in vitro. However, the only known genetic element carrying enterotoxin genes among CoNS is the composite *S. epidermidis* pathogenicity island, SePI. First described in a human *S. epidermidis* FRI909 isolate, SePI carries the orthologs of *sec* and *sel* of *S. aureus* encoding SEC and SEL, respectively [18]. However, efforts to mobilize composite SePI from the *S. epidermidis* FRI909 strain remain ineffective [18].

Numerous PCR-based studies aiming to screen for SE genes report a variable, often frequent occurrence of these genes in CoNS. In turn, studies searching for SE genes based on genome sequence analysis failed to detect SE genes in CoNS, except in some *S. epidermidis* and coagulase-variable *S. hyicus* and *S. agnetis* isolates [19,20,21,22]. We assumed that the SE genes detected in some CoNS strains may occur in their cells in stable and transient forms, the last likely representing a step in SE transfer to CoNS. Therefore, our goal was to assess the origin, stability, and abundance of the DNA carrying SE genes in CoNS isolates obtained from food products and farm animals. We also determined the genomic context of these genes and the expression of CoNS enterotoxins.

## 2. Results

### 2.1. Identification of Enterotoxigenic Coagulase-Negative Staphylococci in Food Products and Livestock Animals

We found amplicons corresponding to the genes of staphylococcal enterotoxins *sea–sed* and *seh* in 29 CoNS isolates (Table 1). The amplicon corresponding to the *sea* gene was found in the *S. saprophyticus* isolate (*n* = 1), *seb* in the *S. xylosus* isolate (*n* = 1), and *sec* in isolates of *S. epidermidis* (*n* = 3) and *S. pasteuri* (*n* = 3), whereas *seh* was found in *S. saprophyticus* (*n* = 1), *S. epidermidis* (*n* = 5), *S. warneri* (*n* = 1), *S. vitulinus* (*n* = 1), *S. chromogenes* (*n* = 2), *S. borealis* (*n* = 7), and *S. haemolyticus* (*n* = 4) (Table 2).

The enterotoxigenic CoNS were isolated from 14/34 (41%) samples of raw cow’s milk. Seven of them were *S. borealis*, four were *S. haemolyticus*, and three were *S. epidermidis* isolates, all displaying the presence of the *seh* gene. In 9 of 92 (10%) meat products, 12 enterotoxigenic CoNS were found, i.e., four *S. epidermidis* isolates harboring *sec* (*n*= 3) and *seh* (*n* = 1) genes, three *S. pasteuri* isolates harboring the *sec* gene, two *S. saprophyticus* isolates harboring *sec* (*n* = 1) and *seh* (*n* = 1) genes, one *S. xylosus* isolate containing the *seb* gene, and one *S. warneri* and one *S. vitulinus* isolate, both containing the *seh* gene. From 50 swine nasal swabs, three enterotoxigenic CoNS strains were obtained (6%), i.e., two *S. chromogenes* and one *S. epidermidis* isolate, all displaying the presence of the *seh* gene.

Amplicons obtained from CoNS isolates corresponding in size to *sea*, *seb*, *sec*, and *seh* products of the reference *S. aureus* strains were sequenced (Genomed S.A., Warsaw, Poland). Then, using the BLAST algorithm, their sequences were compared with the sequences available in the GenBank. We noted that the nucleotide sequences of the *sea*, *seb*, and *seh* amplicons obtained from the DNA of CoNS were homologous to the corresponding genes of known *S. aureus* enterotoxins. The sequence identity was 100% for *sea* and *seb* and 99.72–100% for *seh*. The *sec* amplicons found in *S. epidermidis* and *S. pasteuri* isolates had sequences 100% consistent with the *sec* sequence in *S. epidermidis* FRI909 and *S. epidermidis* 4S [13,18] (Table 3).

Observation of electrophoresed SE amplicons in CoNS showed that the intensity of a significant portion of them (*n* = 26) was lower than the intensity of the corresponding amplicons obtained from the reference *S. aureus* or *S. epidermidis* strains. Only in the case of three enterotoxigenic *S. epidermidis* isolates was the intensity of *sec* amplicons comparable to that of the corresponding reference *S. aureus* and *S. epidermidis* strains. To explore the reasons for this observation, we determined the relative copy numbers of the SE genes in all tested CoNS isolates.

### 2.2. Determination of the Relative Copy Numbers of SE Genes in CoNS Isolates

In all CoNS isolates in which the presence of amplicons corresponding to the enterotoxin genes was detected by PCR, we assessed the relative number of copies of SE genes in genomic DNA.

We showed that in the DNA of 26 enterotoxigenic CoNS, the relative copy number of the SE genes was multiple orders of magnitude lower than in the DNA of the appropriate reference *S. aureus* or *S. epidermidis* strains (Table 4).

In *S. saprophyticus* 5–8 isolate, the relative copy number of the *sea* gene was 622,702 times lower than in the reference *S. aureus* strain. In *S. xylosus* WS08.1 isolate in which the *seb* gene was found, the relative copy number of this gene was 503,324 times lower than in the reference *S. aureus* strain. In *S. pasteuri* 65.1 and 34.1.3 isolates, the relative copy number of the *sec* gene was 3974 and 5980 times lower than in *S. aureus* FRI913, and 4568 and 6873 times lower than in *S. epidermidis* 4S, respectively. In *S. pasteuri* 34.30 isolate with the confirmed *sec* amplicon, the sensitivity of qPCR turned out to be insufficient to evaluate the copy number of the gene. The relative copy number of the *seh* gene was determined in 21 CoNS isolates. In 10 of them, the determination of the number of *seh* copies was below the qPCR sensitivity. In the remaining 11 CoNS, the relative number of the *seh* gene copies was 1050 to 3,461,012 times lower than in *S. aureus* FRI137.

In three *S. epidermidis* isolates of food origin, i.e., *S. epidermidis* 29, 34, and 65.2, *sec* amplicons were detected. Sequences of these amplicons were 100% identical to the sequence of the *sec* gene of *S. epidermidis* 4S and *S. epidermidis* FRI909. The relative copy numbers of the *sec* gene in *S. epidermidis* 29, 34, and 65.2 isolates were from 1.5 times lower to 1.8 times higher than the *sec* copy number in the reference *S. aureus* strain FRI913 and from 1.7 times lower to 1.5-fold higher than the *sec* copy number of *S. epidermidis* 4S strain. The relative copy numbers of the *sec* gene were comparable to *S. aureus* and *S. epidermidis* reference strains only in those enterotoxigenic CoNS.

### 2.3. Determination of Stability of Staphylococcal Enterotoxin Genes

We found that in *S. xylosus* WS08.1 isolate, in which the *seb* gene amplicon was identified, the relative number of enterotoxin gene copies decreased to values not measurable by qPCR after one passage. In *S. saprophyticus* 5–8 isolate, positive for the *sea* gene amplicon, the relative number of copies of this enterotoxin gene varied in the range of 0.0000062828 to 0.0000003002 throughout five consecutive passages. In *S. pasteuri* 65.1 isolate, in which the *sec* gene amplicon was found, the relative number of copies of this enterotoxin gene varied from 0.0000005755 to values not detectable by qPCR throughout five consecutive passages. In the isolate of *S. epidermidis* 275lp, in which the amplicon of the *seh* gene was found, the relative copy number of this gene varied in the range of 0.0000563722 to 0.0000003596 throughout 5 successive passages (Table 5).

Of the 3 *S. epidermidis* isolates in which the relative *sec* copy numbers were comparable to those found in the reference *S. aureus* and *S. epidermidis* strains, *S. epidermidis* 29 isolate was selected for subsequent passages on microbial medium. As a result of five consecutive passages, the relative copy number of the *sec* gene ranged from 0.69 to 1.08.

### 2.4. Mixed Culture of Bacterial Isolates and Incubation of Bacterial Culture with Genomic DNA

The strains of *S. epidermidis* 65.2 and *S. pasteuri* 65.1 were isolated from a sample of a single food product. In both isolates, the amplicons corresponding to the *sec* gene were found by PCR. *S. epidermidis* 65.2 isolate carried *sec* at a high copy number (0.62) while *S. pasteuri* 65.1 at a low copy number (0.0001387956). *S. epidermidis* 65.2 maintained the relative copy number of the *sec* gene at a constant level after five consecutive passages in the microbial medium. In turn in the *S. pasteuri* 65.1 isolate, complete loss of the amplicon corresponding to the *sec* gene was observed after six passages. We assessed the possibility of restoring the *sec* gene in *S. pasteuri* 65.1 by its co-culture with the enterotoxigenic *S. epidermidis* 65.2 isolate. Prior to this experiment, one *S. pasteuri* 65.1 colony was subjected to six passages on an agar plate, which resulted in a complete loss of the PCR signal for the *sec* gene. After a 2-day co-culture of the isolates, we observed the *sec* amplicon in 0–1 of 20 colonies of tested *S. pasteuri* 65.1 isolate. After 5 and 8 days of culture, the *sec* amplicon was noted in 2–4 and 3–4 of 20 examined colonies of S. pasteuri 65.1, respectively.

In all *S. pasteuri* 65.1 colonies derived from co-culture with *S. epidermidis* 65.2, we determined the relative copy number of the *sec* gene by qPCR (Table 6). It was found that the relative copy number of the *sec* gene in all studied colonies was 263,790–5,187,877 times lower than in the reference *S. aureus* strain FRI913, from 298,063 to 5,861,914 times lower than in the *S. epidermidis* 65.2 isolate and from 40 to 779 times lower than that determined in natural *S. pasteuri* 65.1 isolate when isolated from food.

Two selected *S. pasteuri* colonies denoted as II7 and II8, which acquired the *sec* amplicon after the 5-day co-culture with *S. epidermidis* 65.2, were subjected to 5 consecutive passages in the microbial medium. After each passage, we assessed the signal for the *sec* gene by PCR. The presence of the *sec* signal ceased to be PCR-detectable after the third and fourth passage for bacteria derived from colonies II7 and II8, respectively.

To evaluate the DNA containing enterotoxin genes persistence possibility in the culture of CoNS, *S. pasteuri* 65.1 isolate was incubated with the genomic DNA extracted from *S. epidermidis* 65.2. Prior to the experiment, one *S. pasteuri* 65.1 colony was subjected to six consecutive passages in the microbial medium until the complete disappearance of the signal for the *sec* gene. After two days of incubation of *S. pasteuri* 65.1 with a solution of *S. epidermidis* 65.2 genomic DNA, the *sec* gene was detected in 5 out of 8 examined *S. pasteuri* colonies. After 5 and 8 days of incubation, we confirmed the presence of the *sec* gene in 7 and 2 out of 8 examined *S. pasteuri* colonies, respectively.

For all *S. pasteuri* 65.1 colonies that displayed the presence of the *sec* gene after incubation with *S. epidermidis* 65.2 DNA, the relative copy number of the gene was determined by qPCR. In all colonies studied, the relative copy number of the *sec* gene was 12,864 to 355,068 times lower than in the reference strain of *S. aureus* FRI913, from 14,535 to 401,200 times lower than in *S. epidermidis* 65.2 and from 2 to 53 times lower than determined in the natural isolate of *S. pasteuri* 65.1 after isolation from food (Table 7).

### 2.5. Genome Sequencing and Analysis of Selected Bacterial Isolates

The genomic sequence of the enterotoxigenic *S. pasteuri* 65.1 isolate carrying a low-copy-number, unstable *sec* gene was determined. The isolate derived from the same food product as *S. epidermidis* 65.2, carrying a high-copy-number, stable *sec* gene. The number of contigs in the genomic sequence of *S. pasteuri* 65.1 was 146 and the sequence length was 2,618,483 bp (Table 8).

Using the Genome Comparator module of the BIGSdb platform [23], we screened the genomic sequence of *S. pasteuri* 65.1 for the *sec* gene previously detected in this isolate by PCR. We did not identify the *sec* gene in the genomic sequence of *S. pasteuri* 65.1 isolate. The gene was previously documented in *S. epidermidis* 65.2 genome [21].

### 2.6. Production of SEs by CoNS Isolates

We checked the ability to produce SEs in selected CoNS isolates in which *sea*, *seb*, *sec*, and *seh* amplicons were detected by PCR. SEA production was determined in the *S. saprophyticus* 5–8 isolate, SEB in *S. xylosus* WS08.1, SEC and SEL in the *S. pasteuri* 65.1, *S. epidermidis* 65.2, *S. epidermidis* 29, and *S. epidermidis* 34 isolates. We assessed the SEH production in the *S. saprophyticus* 51.2 and *S. epidermidis* 54.1 isolates. Appropriate *S. aureus* reference strains were used as positive controls and the *S. epidermidis* 4S strain was used as a control for SEC and SEL. Negative controls consisted of reference *S. aureus* strains that did not produce specific enterotoxins (Table 9). Cultures of the above-mentioned isolates were carried out in BHI broth. At 6 and 24 h, the number of bacteria was determined, and the concentration of appropriate enterotoxins was measured using ELISA in post-culture liquids.

The number of bacteria ranged from 4.3 log CFU/mL for *S. saprophyticus* 5–8 to 9.4 log CFU/mL for *S. aureus* CCM5757 and *S. aureus* FRI913 after 6 h of cultivation, and from 8.7 log CFU/mL for *S. aureus* FRI913 and *S. xylosus* WS08.1 to 10.0 log CFU/mL for *S. aureus* FRI137 after 24 h of cultivation (Table 10). The only CoNS isolates able to produce SEs were the *S. epidermidis* 29, 34, and 65.2 isolates, all of which secreted SEC and SEL. *S. epidermidis* 29 produced SEC at 476–1180 ng/mL and SEL at 58–223 ng/mL, *S. epidermidis* 34 produced SEC at 337–933 ng/mL and SEL at 18–76 ng/mL, while S. *epidermidis* 65.2 produced SEC at 235–1023 ng/mL and SEL at 17–20 ng/mL after 6 and 24 h of culture, respectively. In the post-culture liquids obtained from the *S. saprophyticus* 5–8, *S. xylosus* WS08.1, *S. pasteuri* 65.1, *S. saprophyticus* 51.4, and *S. epidermidis* 54.1 isolates, no SEs were detected (Table 10).

## 3. Discussion

### 3.1. Origin, Stability, Abundance, and Genomic Context of CoNS Enterotoxin Genes

Enterotoxins produced by staphylococci are the cause of staphylococcal food poisoning. Undoubtedly, *Staphylococcus aureus* is the main producer of these agents [1]. However, over the years, there have been numerous reports confirming the enterotoxigenicity of CoNS isolated from food and animal sources [24,25]. Researchers confirmed the presence of genes encoding enterotoxins orthologous to *S. aureus* SEC and SEL in the genome of the *S. epidermidis* strain FRI909 [18]. The number of reports on the enterotoxigenic CoNS is significant [9,10,13,26,27], but certain research limitations prevent a reliable assessment of this problem. A number of studies conducted so far, showing the presence of amplicons corresponding to enterotoxin genes using PCR, have not confirmed the production of enterotoxins by CoNS at the protein level. Furthermore, sequencing of the obtained SE amplicons and determination of the whole-genome sequence of enterotoxigenic CoNS are rarely performed.

We characterized CoNS isolates from food, cow’s milk, and farm animals, in which we demonstrated the presence of amplicons corresponding to the SE genes by PCR. Sequence analysis of the *sea*, *seb*, *sec*, and *seh* amplicons in CoNS isolates showed that they correspond to the sequences of known enterotoxins of *S. aureus* or *S. epidermidis.* The signal strength of the majority of these amplicons after electrophoretic separation was weaker than for the SE amplicons of *S. aureus*. The exception was the *sec* gene amplicons in three isolates of *S. epidermidis*, which harbor the *sec* gene.

We have shown that in a significant part of enterotoxigenic CoNS isolates, the relative number of SE gene copies was much lower than in *S. aureus* strains. The only isolates with relative copy numbers of SE genes comparable to the reference strains of *S. aureus* and *S. epidermidis* 4S strain described earlier [13] were three isolates of *S. epidermidis* in which we detected the *sec* gene by PCR. We quantified the SE signals referring them to the *tufA* gene known to occur in a single copy in CoNS cells, allowing to indirectly compare the number of SE copies to the number of CoNS cells. Based on our research it can be interpreted that in CoNS isolates carrying the SE genes at low copy numbers a given SE gene occurs in one bacterial cell per thousands or millions non-toxigenic cells.

Our results also indicate that CoNS isolates in which the relative number of enterotoxin genes is significantly lower than in *S. aureus* strains may lose the PCR signal of the SE genes. The phenomenon of the loss of genetic elements containing SE genes by CoNS during culture in microbial media may suggest that SE genes occur in CoNS in an unstable form, and bacteria can lose them during cell division. Alternatively, this observation may indicate a continuous transmission of elements containing SE genes from staphylococci that harbor stable enterotoxin genes to the CoNS, which may occur in the environment. Isolation of the CoNS from the environment and further cultivation under laboratory conditions would break the horizontal gene-transfer process. This scenario seemed probable when we isolated the *S. epidermidis* 65.2 strain displaying a stable *sec* gene in a high copy number, together with the *S. pasteuri* 65.1 strain with the unstable *sec* gene present in a low copy number from a single food product.

To determine whether the *sec* gene is integrated into the genome of *S. pasteuri* 65.1, whole-genome sequencing of the natural *S. pasteuri* 65.1 isolate was performed. Our analysis did not show the presence of the *sec* gene or the elements of the composite SePI in the whole genomic sequence of the *S. pasteuri* 65.1 isolate. According to current knowledge, the *sec* gene is part of certain SaPIs and the *S. epidermidis* composite SePI. Researchers have convincingly described the possibility of mobilization and horizontal SaPI transfer involving helper phages between *S. aureus* strains and between *S. aureus* and other bacterial species in vitro [14,15,16,17]. Attempts to mobilize the helper phage Φ909 in *S. epidermidis* FRI909 strain and transfer the SePI element by Madhusoodanan et al. [18] did not have any effect. This may suggest that the composite SePI element has no potential for transmission between *S. epidermidis* and other microorganisms.

To determine the possibility of horizontal transfer of genetic elements containing enterotoxin genes between CoNS species, we performed a joint culture of *sec*-positive staphylococci isolated from the same food product, i.e., *S. epidermidis* 65.2 and *S. pasteuri* 65.1. The unstable *sec* gene in the natural isolate of *S. pasteuri* 65.1, derived from the food product, was eliminated in the process of subsequent passages in the microbiological medium. During the co-culture of *S. epidermidis* 65.2 and *S. pasteuri* 65.1, we observed the appearance of amplicons corresponding to the *sec* gene in some of the *S. pasteuri* colonies. The relative copy number of the *sec* gene acquired by *S. pasteuri* 65.1 was at a level corresponding to the low copy number of enterotoxin genes in the CoNS that possess unstable enterotoxin genes. Further passages of selected *S. pasteuri* 65.1 colonies that acquired *sec* resulted in a gradual disappearance of the *sec* amplicon up to its complete loss, as previously seen in the natural isolate of *S. pasteuri* 65.1 isolated from the food product. The results obtained indicate that elements containing staphylococcal enterotoxin genes can be transferred in the food environment between CoNS species.

Given that the presence of the *sec* gene or any of the SePI elements has not been identified in the complete genome of the natural *S. pasteuri* 65.1 isolate, we assumed that the observed acquisition of *sec* by *S. pasteuri* may not occur due to the transfer of the SePI element containing the *sec* gene to *S. pasteuri* cells, but only as a result of the persistence of extracellular DNA of *S. epidermidis* 65.2 in the culture of the *S. pasteuri* isolate. We evaluated this possibility by incubating the *S. pasteuri* 65.1 culture with genomic DNA from *S. epidermidis* 65.2. The results of this experiment indicate the probability of persistence of, most likely extracellular, DNA on the surface of CoNS cells. This could mean that the observed in vitro transfer of the *sec* gene between *S. epidermidis* and *S. pasteuri* was not the result of the mobilization of the genetic element and its horizontal transfer. The results obtained indicate that the generated amplicons corresponding to the sequences of SE genes from CoNS isolates may be associated with the long-term persistence of DNA containing enterotoxin genes in cultures of these bacteria.

The genomes of enterotoxigenic *S. epidermidis* 29, 34, and 65.2 strains isolated from food were already sequenced and analyzed for their SE gene contents and their genomic context [21]. These strains produced stable signals for PCR-identified SE genes with high copy numbers comparable to reference enterotoxigenic *S. aureus* and *S. epidermidis* strains. We identified genes encoding *S. aureus* SEC and SEL orthologs in the genomic sequences of the 29, 34, and 65.2 *S. epidermidis* strains. Genes encoding these orthologs were located in a composite genomic-island SePI that had the same genomic integration site in each *S. epidermidis* strain [21].

### 3.2. Food Safety Hazards and Clinical Relevance of CoNS Enterotoxins

To assess food safety hazards related to enterotoxigenic CoNS, we determined the ability to produce staphylococcal enterotoxins by selected isolates of these bacteria. Based on the results obtained, we can assume that the only CoNS isolates capable of SE production are those in which enterotoxin genes are present in a stable form in high copy numbers. Isolates of CoNS in which enterotoxin genes in an unstable form and low copy numbers are present are not capable of producing enterotoxins, even under optimal laboratory conditions. The enterotoxigenicity of staphylococci contributed to a number of human and animal diseases, including organ and systemic disorders [28,29]. However, to date, all these cases were only associated with SE-producing *S. aureus*. There are scarce data on the correlation between the enterotoxigenicity of CoNS and its clinical importance. Currently available WGS-based research indicates that other virulence factors, such as genes encoding adhesion factors, biofilm production, hemolysins, and exoenzymes, may affect the pathogenicity of CoNS [30]. We recently found that SE produced by *S. epidermidis* modifies the intestinal immune response and contributes to gut damage in mice [31].

### 3.3. Conclusions

We demonstrated that enterotoxin genes can occur at highly variable copy numbers in CoNS. However, we could identify enterotoxin genes only in the whole-genome sequences of CoNS that carried them in high numbers of copies. Only those enterotoxins were expressed at the protein level. The enterotoxin genes appearing in CoNS isolates at low copy numbers may be a result of a transient transfer of DNA containing enterotoxin genes from co-occurring enterotoxigenic staphylococci. Our results indicate that the detection of enterotoxin genes based on PCR in CoNS should always require an additional control, such as analysis of the presence of the SE gene in the bacterial genome or detection of the SE protein. Our survey also revealed *S. epidermidis* to be a CoNS species harboring SE genes in a stable form in the bacterial chromosome and expressing them as a protein.

## 4. Materials and Methods

### 4.1. Bacterial Strains’ Isolation and Staphylococcal Species’ Identification

In this research, 141 food products (92 meat products, 34 samples of raw cow’s milk, and 15 samples of rennet cheese) and 50 swine nasal swabs collected in a slaughterhouse were screened for the presence of enterotoxigenic coagulase-negative staphylococci. Assuming that enterotoxigenic CoNS may enter the food chain from animal sources, food samples represented products obtained from raw material not processed thermally during production. Similarly, milk was non-processed, and swine swabs were taken from animals just after stunning and bleeding.

A food sample (5 ± 1 g) or a swab was placed in 20 mL of Giolitti-Cantoni broth (Merck, Darmstadt, Germany). After 24–48 h of incubation at 37 °C, positive samples (blackening of the medium or a gray-black precipitate) were streaked onto Baird-Parker agar (Merck, Darmstadt, Germany) and incubated for another 48 h. Next, a loopful of biomass from the plate was transferred into 5 mL of brain-heart-infusion broth (BTL, Warsaw, Poland) and incubated for 18–24 h at 37 °C. From these cultures, DNA was isolated to identify the presence of *sea*–*sed* or *seh* genes. In case of a positive result, separate bacterial colonies were screened for the presence of amplicons of the corresponding staphylococcal enterotoxins. To exclude contamination with enterotoxigenic *S. aureus*, the CoNS DNA was inspected for coagulase (*clf*) and nuclease (*nuc*) genes. Species identification of enterotoxigenic CoNS isolates was performed by analyzing fragments of the *dnaJ* or *tufA* gene sequences and their comparison with the GenBank database resources.

### 4.2. Genomic DNA Extraction

A 2 mL sample of overnight bacterial culture in BHI broth (BTL, Warsaw, Poland) was centrifuged at 12,000× *g* for 5 min. The bacterial pellet was suspended in 150 μL of 0.1 M Tris-HCl buffer, pH 7.4, containing 2 U of lysostaphin (A&A Biotechnology, Gdańsk, Poland), and incubated for 30 min at 37 °C. Next, 15 μL of 10% SDS was added and incubated for 15 min at 37 °C, and then 200 μL of 5 M guanidine hydrochloride solution was added and left for 10 min at room temperature. The DNA was extracted by phenol and chloroform, precipitated by ethanol, dissolved in 50 μL of UltraPure™ Distilled Water (Thermo Fischer Scientific Inc., Waltham, MA, USA) and stored at −20 °C.

### 4.3. PCR Amplification

The tested isolates were screened for the presence of staphylococcal enterotoxin *sea*, *seb*, *sec*, *sed*, and *seh* genes using PCR. Each reaction mixture (25 μL) consisted of 2.5 μL 10× DreamTaq Buffer (Thermo Fischer Scientific Inc., Waltham, MA, USA), 0.5 μL of 10 mM dNTPs mix (Thermo Fischer Scientific Inc., Waltham, MA, USA), 0.1 μL of each 10 μM primer, 0.5 U of DreamTaq polymerase (Thermo Fischer Scientific Inc., Waltham, MA, USA), and 1 μL of genomic DNA. Reference *S. aureus* strains FRI913, CCM5757, FRI1151m, and FRI137 were used as PCR controls (Table 11). All amplicons corresponding to *S. aureus* enterotoxin genes were sequenced (Genomed S.A., Warsaw, Poland). The presence of *clf* and *nuc* genes was determined by duplex PCR. The composition of the reaction mixture was the same as described above. All primers used in this study and all PCR protocols are listed in Supplementary Table S1.

### 4.4. Determination of Relative Gene Copy Numbers in the Tested Staphylococcal Isolates

In all isolates of enterotoxigenic CoNS, the relative copy number of enterotoxin genes was determined by quantitative PCR (qPCR) using a CFX Connect™ thermal cycler (Bio-Rad Laboratories Inc., Hercules, CA, USA). The *tufA* gene was selected as the reference gene for signal normalization for enterotoxin genes. This gene is present in the genomes of CoNS species in one copy per genome. The primers used for SE genes’ amplification were the same as those used in PCR, and the primers used for amplification of the housekeeping gene were as follows: tuf2-F 5′-GACTACGCTGAAGCTGGTGAC-3′ and tuf3-R 5′-AGTACGGAAATAGAATTGTGG-3′. The reaction mixture (20 µL) consisted of 10 µL of KAPA SYBR^®^ FAST qPCR Master Mix (2X) (Sigma-Aldrich Co., Saint Louis, MO, USA), 0.8 µL of each 1.25 µM primer, and 100 ng of genomic DNA. The qPCR amplification conditions included: initial denaturation at 95 °C for 10 min, followed by 40 cycles of amplification (95 °C for 15 s, 55 °C for 20 s, and 72 °C for 40 s). PCR specificity was monitored by analyzing the melting curves of PCR products in a temperature range from 90 °C to 56 °C. For each primer pair, PCR efficiencies were determined by running serial five-fold dilutions of the template. Bio-Rad CFX Manager 3.1 software (Bio-Rad Laboratories Inc., Hercules, CA, USA) was used for the calculations. The Ct values of a given enterotoxin gene were normalized to the Ct of the housekeeping *tufA* gene, taking into calculation the amplification efficiency of the gene, using the normalized expression method (ΔΔCt) [32]. Then, the values of the relative copy number of each enterotoxin gene were calculated by referring them to the sample showing the lowest normalized expression value, which was assigned the value of 1. Therefore, the values of the relative copy numbers of a given enterotoxin gene showed the copy numbers of that gene relative to the arbitrarily chosen lowest value. Each qPCR run consisted of two replicates for each sample.

### 4.5. Determination of Stability of Enterotoxin Genes in CoNS Isolates

In SE gene-positive CoNS isolates, the stability of these genes was determined. Bacteria were streaked onto BHI agar (BTL, Warsaw, Poland) and incubated overnight at 37 °C. A single, well-separated bacterial colony was transferred to a fresh medium every 24 h. Two to five subsequent passages were carried out. During each passage, a portion of the selected bacterial colony was transferred into BHI broth for genomic DNA isolation. DNA obtained from subsequent bacterial passages was tested by qPCR to determine the relative copy numbers of SE genes, as described above.

### 4.6. Mixed Culture of Bacterial Isolates and Incubation of Bacterial Culture with Genomic DNA

To investigate the possibility of transfer of genetic elements carrying enterotoxin genes between CoNS isolates, the enterotoxigenic *S. epidermidis* 65.2 strain containing a stable *sec* gene of high relative copy number was cultured together with the *S. pasteuri* 65.1 strain, in which the unstable, low-copy *sec* gene was lost as a result of consecutive passages in a microbial medium. The co-culture was conducted as described before by McCarthy et al. [33] with minor modifications. Serial dilutions of the samples were plated on MSA medium (BTL, Poland) and incubated at 37 °C for 24–48 h. The medium allowed the distinction between *S. epidermidis* and *S. pasteuri* based on the colony morphology (yellow versus white colonies). The culture was continued for eight days with sampling after two, five, and eight days. At each time point, 20 well-separated colonies of *S. pasteuri* 65.1 were collected, transferred into 5 mL of BHI broth, and incubated overnight at 37 °C with agitation at 230 rpm. For each sample, the DNA was extracted to determine the relative copy number of the *sec* gene. Two selected colonies of *S. pasteuri* 65.1, positive for the *sec* gene after co-culture with *S. epidermidis* 65.2, were subjected to five consecutive passages on an agar medium to assess the stability of the gene.

To evaluate the persistence of extracellular DNA containing enterotoxin genes in CoNS cell culture, the *S. pasteuri* 65.1 isolate, in which the unstable *sec* gene was lost, was incubated with genomic DNA of *S. epidermidis* 65.2. To do so, 6 mL of BHI broth was inoculated with a single colony of *S. pasteuri* 65.1 and incubated overnight at 37 °C with agitation at 80 rpm. Next, 60 μL of diluted bacterial culture (OD_600_ = 0.03) was transferred into 6 mL of fresh BHI medium containing 30 μg of *S. epidermidis* 65.2 strain genomic DNA (*sec* +). The amount of genomic DNA added was determined based on the extraction of extracellular DNA from *S. epidermidis* 65.2 post-culture liquid. *S. pasteuri* 65.1 was cultured for eight days under the conditions described above. Bacteria were diluted daily by transferring 60 μL of the culture to 6 mL of fresh medium. After two, five, and eight days, samples of 100 μL were taken, and their serial dilutions were plated on BHI agar (overnight incubation at 37 °C). After that, eight colonies were selected for qPCR.

### 4.7. Growth Curve Determination and Sandwich ELISA of Selected Bacterial Isolates

A single bacterial colony was inoculated in 5 mL of BHI broth and incubated overnight at 37 °C with agitation at 230 rpm. Then, the bacterial suspension was diluted to an OD_600_ of 0.03 in 100 mL of BHI; the culture was carried out at 37 °C with agitation at 230 rpm for 24 h. Samples for the cell count and ELISA were collected at 6 and 24 h of culture. Bacterial cells were quantified by plating serial dilutions of the culture on BHI agar. At each time point, 2 mL of bacterial culture sample was centrifuged at 12,000× *g* for 5 min, and 1.5 mL of supernatant was collected and stored at −20 °C until analyzed. To neutralize the A protein, 100 µL of each sample was incubated with the final concentration of 20% rabbit serum (IBSS Biomed S.A., Kraków, Poland) overnight at 4 °C.

Native SEA (Sigma-Aldrich Co., Saint Louis, MO, USA), recombinant rSEC [34], and rSEH [35] were used to determine the standard curve for the ELISA. Recombinant rSEL was obtained as described by Omoe et al. [36]. The coding region of the mature *sel* gene from *S. aureus* FRI913 was amplified by PCR and the amplicons were digested with NcoI and XhoI (Thermo Fischer Scientific Inc., Waltham, MA, USA). The digested amplicon was cloned into a pET-22b plasmid vector (Merck, Darmstadt, Germany), introduced into NovaBlue *Escherichia coli* cells (Merck, Darmstadt, Germany), and next transformed into *E. coli* Rosetta-gami DE3 cells (Merck, Darmstadt, Germany). The SEL expression was performed using the IPTG induction (Merck, Darmstadt, Germany). Purification of rSEL was performed on His-Select cobalt affinity gel (Merck, Darmstadt, Germany), with on-column refolding. The purity of rSEL preparation was checked with SDS-PAGE. Polyclonal rabbit anti-SEA (OriGene Technologies, Inc., Rockville, MD, USA), anti-SEC (OriGene Technologies, Inc., Rockville, MD, USA), and anti-SEH (Abcam, Cambridge, UK) antibodies were used to detect SEA, SEC, and SEH enterotoxins. Anti-SEL sera were obtained by immunizing rabbits with 30 µg of rSEL using the protocol described by Lis et al. [35]. Immunization was repeated four times at three-week intervals. Fourteen days after the last immunization, the animals were bled and the serum was prepared. The antibody titer was monitored by indirect ELISA. Monospecific polyclonal anti-SEL antibodies were obtained by affinity purification of the sera with rSEL coupled to CNBr-activated Sepharose 4B (Sigma-Aldrich Co., Saint Louis, MO, USA). The specificity of the antisera was tested by Western blotting, using culture supernatants of enterotoxigenic, *sel*-negative *S. aureus* strains FRI1151m and CCM5757, and the *sel*-positive strains FRI913 and FRI137.

Antibodies conjugated with biotin N-hydroxy succinimide ester (Sigma-Aldrich Co., Saint Louis, MO, USA) were used as secondary antibodies. These conjugates were obtained by incubating samples of anti-SEA, anti-SEC, and anti-SEL sera with a solution of biotin hydroxysuccinylimide ester [35]. A solution of horseradish peroxidase streptavidin conjugate was used to detect biotinylated anti-SEA, anti-SEC, and anti-SEL antibodies. As a secondary serum for the detection of SEH enterotoxin, an anti-SEH horseradish peroxidase conjugate was used (Abcam, Cambridge, UK). ELISA assay was performed as described earlier by Schubert et al. [34]. For the detection of the SEB enterotoxin, the RIDASCREEN^®^ SET Total kit (R-Biopharm AG, Germany) was used to qualitatively detect staphylococcal enterotoxins (A–E) according to the manufacturer’s instructions. The enterotoxin concentration was measured with SEA, rSEC, rSEH, and rSEL as standards, using a four-parameter logistic curve. Plotting standard curves and calculating enterotoxin concentrations was performed using GraphPad Prism 8 software (GraphPad Software, San Diego, CA, USA). All experiments were carried out in duplicate with two biological replicates.

### 4.8. Genome Sequencing and Analysis of Bacterial Isolates

Enterotoxigenic *S. pasteuri* 65.1 was subjected to NGS sequencing. Genomic DNA was extracted with the MasterPure™ kit (Lucigen, Middleton, WI, USA) according to the manufacturer’s instructions and measured using a DeNovix spectrophotometer (DeNovix Inc., Wilmington, DE, USA). De novo whole-genome sequencing was performed using MiSeq (Illumina, San Diego, CA, USA) in the PE250 mode (Genomed S.A., Warsaw, Poland). DNA libraries were prepared using the NEBNext^®^ DNA Library Prep Master Mix Set for Illumina^®^ (New England Biolabs, Ipswich, MA, USA). The obtained readings were cut with Cutadapt 1.16 [37]. De novo assembly was performed using the SPAdes algorithm 3.11.1 [38]. The obtained contigs were ordered using Mauve by aligning them to the sequence of the complete genome of the *S. pasteuri* SP1 (accession number CP004014) isolate available in the GenBank database. The whole genome sequence of the isolate was deposited in the BIGSdb database [23] and analyzed for the presence of genes of 23 known staphylococcal enterotoxins obtained from the GenBank database using the Genome Comparator module of the BIGSdb platform. The genome sequence of *S. pasteuri* 65.1 has been deposited in the DDBJ/ENA/GenBank under the accession number JAJTJN000000000. The version described in this paper is version JAJTJN010000000.

## Figures and Tables

**Table 1:** Bacterial isolates used in this study.

No.	Isolate	Species	Origin
1.	5–8	*S. saprophyticus*	smoked meat
2.	WS08.1	*S. xylosus*	maturing sausage
3.	29	*S. epidermidis*	jamón ibérico ham
4.	34	*S. epidermidis*	dried sausage with wild boar meat
5.	34.1.3	*S. pasteuri*	dried sausage with wild boar meat
6.	34.30	*S. pasteuri*	dried sausage with wild boar meat
7.	65.2	*S. epidermidis*	dried sausage with wild boar meat
8.	65.1	*S. pasteuri*	dried sausage with wild boar meat
9.	51.2	*S. saprophyticus*	Schwarzwald ham
10.	54.1	*S. epidermidis*	fuet sausage
11.	70.1	*S. warneri*	smoked meat
12.	72.3	*S. vitulinus*	dried sausage
13.	210.1	*S. epidermidis*	swine nasal swab
14.	210.3	*S. chromogenes*	swine nasal swab
15.	231.4	*S. chromogenes*	swine nasal swab
16.	206D1	*S. epidermidis*	raw cow’s milk
17.	275lp	*S. epidermidis*	raw cow’s milk
18.	424C1	*S. epidermidis*	raw cow’s milk
19.	17B	*S. borealis*	raw cow’s milk
20.	263D	*S. haemolyticus*	raw cow’s milk
21.	325C	*S. haemolyticus*	raw cow’s milk
22.	3A	*S. borealis*	raw cow’s milk
23.	11D2	*S. borealis*	raw cow’s milk
24.	321C	*S. borealis*	raw cow’s milk
25.	424A	*S. borealis*	raw cow’s milk
26.	9541D	*S. borealis*	raw cow’s milk
27.	41B	*S. borealis*	raw cow’s milk
28.	23JK	*S. haemolyticus*	raw cow’s milk
29.	18C	*S. haemolyticus*	raw cow’s milk

**Table 2:** Presence of staphylococcal enterotoxin genes in *Staphylococcus* spp. isolates.

No.	Species	Number of Isolates	Number of Isolates Containing Enterotoxin Genes			
			*sea*	*seb*	*sec*	*seh*
1.	*S. chromogenes*	2	-	-	-	2
2.	*S. epidermidis*	8	-	-	3	5
3.	*S. haemolyticus*	4	-	-	-	4
4.	*S. pasteuri*	3	-	-	3	-
5.	*S. saprophyticus*	2	1	-	-	1
6.	*S. vitulinus*	1	-	-	-	1
7.	*S. warneri*	1	-	-	-	1
8.	*S. xylosus*	1	-	1	-	-
9.	*S. borealis*	7	-	-	-	7

**Table 3:** Presence of staphylococcal enterotoxin genes in studied isolates of CoNS and analysis of their nucleotide sequences.

No.	Isolate	Species	Enterotoxin Genes	BLAST Parameters						Sequence Lenght [bp]
				Max Score	Total Score	Query Cover	E Value	% Identity	Accession Number	
1.	5–8	*S. saprophyticus*	*sea*	640	640	100%	1.00 × 10^−179^	100.00%	LR134093.1	346
2.	WS08.1	*S. xylosus*	*seb*	651	651	99%	0.0	100.00%	CP039157.1	353
3.	29	*S. epidermidis*	*sec*	1480	1480	100%	0.0	100.00%	CP024437.1	812
4.	34	*S. epidermidis*		1480	1480	100%	0.0	100.00%	CP024437.1	812
5.	34.1.3	*S. pasteuri*		1480	1480	100%	0.1	100.00%	CP024437.2	812
6.	34.30	*S. pasteuri*		470	470	99%	1.00 × 10^−128^	100.00%	LR134088.1	255
7.	65.2	*S. epidermidis*		1480	1480	100%	0.0	100.00%	CP024437.1	812
8.	65.1	*S. pasteuri*		390	390	100%	9.00 × 10^−105^	100.00%	CP024437.1	211
9.	51.2	*S. saprophyticus*	*seh*	656	656	99%	0.0	99.72%	AY345144.1	359
10.	54.1	*S. epidermidis*		656	656	99%	0.0	99.72%	AY345144.1	359
11.	70.1	*S. warneri*								
12.	72.3	*S. vitulinus*								
13.	210.1	*S. epidermidis*		662	662	99%	0.0	100.00%	AY345144.1	359
14.	210.3	*S. chromogenes*								
15.	231.4	*S. chromogenes*								
16.	206D1	*S. epidermidis*		645	645	100%	0.0	100.00%	CP034441.1	349
17.	275lp	*S. epidermidis*								
18.	424C1	*S. epidermidis*		664	664	100%	0.0	100.00%	AY345144.1	359
19.	17B	*S. borealis*		645	645	100%	0.0	100.00%	AY345144.1	349
20.	263D	*S. haemolyticus*								
21.	325C	*S. haemolyticus*								
22.	3A	*S. borealis*								
23.	11D2	*S. borealis*								
24.	321C	*S. borealis*								
25.	424A	*S. borealis*								
26.	9541D	*S. borealis*								
27.	41B	*S. borealis*		658	658	100%	0.0	99.72%	CP034441.1	359
28.	23JK	*S. haemolyticus*								
29.	18C	*S. haemolyticus*								

**Table 4:** Relative copy numbers of SE genes.

Gene	Isolate	Species	Relative Gene Copy Number	SD	Mean Ct	SD (Ct)
*sea*	5–8	*S. saprophyticus*	0.0000016059	0.0000003	30.27	0.23
	FRI913	*S. aureus*	1	0.2348314	11.51	0.03
*seb*	CCM5757	*S. aureus*	1	0.1075083	17.5	0.14
	WS08.1	*S. xylosus*	0.0000019868	0.0000004	33.34	0.25
*sec*	34.1.3	*S. pasteuri*	0.0000922401	0.0000958	27.38	1.42
	34.30	*S. pasteuri*	n/d	-	n/d	-
	65.1	*S. pasteuri*	0.0001387956	0.0001296	28.25	1.41
	29	*S. epidermidis*	1	0.2141541	15.26	0.03
	34	*S. epidermidis*	0.3779972882	0.0459499	15.93	0.17
	65.2	*S. epidermidis*	0.6169930460	0.1108341	14.42	0.23
	4S	*S. epidermidis*	0.6340004893	0.1377956	14.36	0.26
	FRI913	*S. aureus*	0.5515770501	0.0360212	17.36	0.02
*seh*	11D2	*S. borealis*	0.0000013360	0.0000005	34.46	0.56
	17B	*S. borealis*	0.0000016867	0.0000006	34.87	0.51
	206D1	*S. epidermidis*	0.0000138600	0.0000025	32.39	0.27
	23JK	*S. haemolyticus*	n/d	-	n/d	-
	263D	*S. haemolyticus*	0.0000002889	0.0000001	33.21	0.37
	275lp	*S. epidermidis*	0.0000106355	0.0000016	31.40	0.17
	325C	*S. haemolyticus*	n/d	-	n/d	-
	3A	*S. borealis*	0.0000026664	0.0000013	33.95	0.71
	424A	*S. borealis*	0.0000219215	0.0000147	33.84	0.90
	424C1	*S. epidermidis*	0.0000271405	0.0000104	32.01	0.47
	51.2	*S. saprophyticus*	0.0009528300	0.0003784	26.52	0.21
	54.1	*S. epidermidis*	0.0006245173	0.0004667	28.31	1.11
	70.1	*S. warneri*	n/d	-	n/d	-
	72.3	*S. vitulinus*	0.0000873649	0.0000553	33.45	0.86
	210.1	*S. epidermidis*	n/d	-	n/d	-
	210.3	*S. chromogenes*	n/d	-	n/d	-
	231.4	*S. chromogenes*	n/d	-	n/d	-
	321C	*S. borealis*	n/d	-	n/d	-
	9541D	*S. borealis*	n/d	-	n/d	-
	41B	*S. borealis*	n/d	-	n/d	-
	18C	*S. haemolyticus*	n/d	-	n/d	-
	FRI137	*S. aureus*	1	0.1672767	13.28	0.15

n/d—non-detectable in qPCR.

**Table 5:** Relative copy numbers of enterotoxin genes in CoNS isolates subjected to consecutive passages on microbiological media.

Gene	Isolate	No. of Passage	Relative Gene Copy Number	SD	Mean Ct	SD (Ct)
*sea*	*S. saprophyticus* 5–8	1	0.0000016059	0.0000003	30.27	0.23
		2	0.0000062828	0.0000009	29.09	0.05
		3	0.0000019716	0.0000003	31.45	0.23
		4	0.0000003002	0.0000001	34.03	0.33
		5	0.0000004322	0.0000001	33.78	0.49
*seb*	*S. xylosus* WS08.1	1	0.0000019868	0.0000004	33.34	0.25
		2	n/d	-	n/d	-
*sec*	*S. pasteuri* 65.1	1	0.0000005755	0.0000001	37.52	0.06
		2	n/d	-	n/d	-
		3	0.0000000322	0.0000001	37.85	1.13
		4	0.0000001303	0.0000001	35.88	0.37
		5	n/d	-	n/d	-
	*S. epidermidis* 29	1	1	0.2141541	15.26	0.03
		2	0.6902912192	0.0312809	13.11	0.03
		3	0.7780831151	0.1391411	16.16	0.04
		4	1.0812638900	0.1415436	11.86	0.04
		5	0.8365744160	0.1012565	13.22	0.18
*seh*	*S. epidermidis* 275lp	1	0.0000563722	0.0000226	31.67	0.53
		2	0.0000061340	0.0000063	34.87	0.85
		3	0.0000127956	0.0000006	34.96	0.06
		4	0.0000003596	0.0000002	35.72	0.89
		5	0.0000028244	0.0000015	34.33	0.71

n/d—non-detectable in qPCR.

**Table 6:** Relative copy number of the *sec* gene determined in colonies of *S. pasteuri* 65.1 co-cultured with *S. epidermidis* 65.2 isolate.

Days of Culture	Colony Symbol	Relative Gene Copy Number	SD	Mean CT	SD (Ct)
2	I 16	0.0000001141	0.00000003	36.31	0.38
5	I 7	0.0000001724	0.0000001	36.70	0.30
	I 8	0.0000018644	0.0000008	34.11	0
	I 10	0.0000002251	0.00000002	36.93	0
	I 13	n/d	-	n/d	-
	II 7	0.0000016132	0.0000014	33.54	1.26
	II 8	0.0000007944	0.0000004	33.54	0.75
8	I 8	0.0000012647	0.0000010	34.25	0
	I 9	0.0000001023	0.00000004	35.74	0.56
	I 10	0.0000002328	0.0000002	34.90	0.11
	II 2	0.0000003608	0.0000004	34.56	1.77
	II 7	0.0000003394	0.00000003	34.03	0.13
	II 8	0.0000001604	0.0000002	35.58	1.46
	II 9	0.0000020119	0.0000007	32.87	0.55

n/d—non-detectable in qPCR.

**Table 7:** Relative copy number of the *sec* gene determined in colonies of *S. pasteuri* 65.1 isolate after incubation with *S. epidermidis* 65.2 isolate DNA.

Days of Culture	Colony Symbol	Relative Gene Copy Number	SD	Mean CT	SD (Ct)
2	1	0.0000014947	0.0000006	33.87	0.57
	4	0.0000043783	0.0000020	31.78	0.71
	5	0.0000079241	0.0000047	31.25	0.80
	6	0.0000023815	0.0000002	32.64	0.06
	7	0.0000011681	0.0000009	34.04	1.16
5	1	0.0000129606	0.0000001	32.05	0.02
	2	0.0000412559	0.0000029	29.56	0.11
	3	0.0000063897	0.0000041	31.71	0.99
	4	0.0000037225	0.0000009	32.51	0.36
	6	0.0000046248	0.0000006	31.86	0.07
	7	0.0000077427	0.0000026	31.18	0.35
	8	0.0000066784	0.0000007	31.55	0
8	1	0.0000085114	0.0000026	32.15	0.46
	4	0.0000023988	0.0000004	33.16	0.19

**Table 8:** Staphylococcal isolates for which genomic sequences have been determined.

No.	Isolate	Species	No. of Contigs	Sequence Length [bp]	Reference
1.	29	*S. epidermidis*	58	2,543,747	[21] *
2.	34	*S. epidermidis*	83	2,558,976	[21] *
3.	65.1	*S. pasteuri*	146	2,618,483	This study
4.	65.2	*S. epidermidis*	48	2,536,954	[21] *

* Genomic sequences of *S. epidermidis* 29 (filename 2056_29), *S. epidermidis* 34 (filename 2055_34) and *S. epidermidis* 65.2 (filename 2428_65) can be found at: https://figshare.com/articles/dataset/Mosaic_ancestry_inter-species_gene_flow_and_genetic_diversity_of_composite_enterotoxigenic_Staphylococcus_epidermidis_pathogenicity_islands_/8168483 (last accessed: 24 February 2022).

**Table 9:** CoNS isolates in which the ability to produce staphylococcal enterotoxins was determined.

Staphylococcal Enterotoxin	Isolates Tested	Positive Control	Negative Control
SEA	*S. saprophyticus* 5–8	*S. aureus* FRI913	*S. aureus* CCM5757
SEB	*S. xylosus* WS08.1	*S. aureus* CCM5757	-
SEC and SEL	*S. epidermidis* 29	*S. aureus* FRI913	*S. aureus* CCM5757
	*S. epidermidis* 34	*S. epidermidis* 4S	
	*S. epidermidis* 65.2		
	*S. pasteuri* 65.1		
SEH	*S. saprophyticus* 51.2	*S. aureus* FRI137	*S. aureus* CCM5757
	*S. epidermidis* 54.1		

**Table 10:** Bacterial growth and staphylococcal enterotoxin production determined by ELISA assay.

SE	Isolate	Bacterial Growth		SE Concentration [ng/mL]	
		[log CFU/ml]			
		6 h	24 h	6 h	24 h
SEA	*S. saprophyticus* 5–8	4.3 ± 0.3	8.8 ± 0.2	0	0
	*S. aureus* FRI913	8.0 ± 0.2	8.7 ± 0.3	36.7 ± 3.9	54.6 ± 9.9
	*S. aureus* CCM5757	9.4 ± 0.3	9.2 ± 0.2	0	0
SEB	*S. xylosus* WS08.1	8.2 ± 0.2	8.7 ± 0.3	-	-
	*S. aureus* CCM5757	9.4 ± 0.3	9.2 ± 0.2	+	+
SEC	*S. epidermidis* 29	9.0 ± 0.1	9.4 ± 0.3	476.1 ± 91.7	1180.0 ± 79.6
	*S. epidermidis* 34	9.2 ± 0.2	9.6 ± 0.1	337.7 ± 17.4	933.8 ± 65.1
	*S. pasteuri* 65.1	8.0 ± 0.1	9.1 ± 0.1	0	0
	*S. epidermidis* 65.2	9.2 ± 0.2	9.5 ± 0.1	235.6 ± 29.3	1023.7 ± 100.5
	*S. epidermidis* 4S	9.3 ± 0.2	9.5 ± 0.2	881.0 ± 59.9	4094.9 ± 559.4
	*S. aureus* FRI913	9.4 ± 0.1	9.7 ± 0.2	1392.7 ± 90.1	2530.9 ± 136.9
	*S. aureus* CCM5757	9.4 ± 0.3	9.2 ± 0.2	0	0
SEH	*S. saprophyticus* 51.2	4.8 ± 0.1	8.9 ± 0.2	0	0
	*S. epidermidis* 54.1	8.7 ± 0.1	9.6 ± 0.1	0	0
	*S. aureus* FRI137	8.6 ± 0.1	10.0 ± 0.1	418.5 ± 72.8	761.0 ± 85.5
	*S. aureus* CCM5757	9.4 ± 0.3	9.2 ± 0.2	0	0
SEL	*S. epidermidis* 29	9.0 ± 0.1	9.4 ± 0.3	57.6 ± 24.1	222.9 ± 58.8
	*S. epidermidis* 34	9.2 ± 0.2	9.6 ± 0.1	17.9 ± 1.9	75.8 ± 17.9
	*S. pasteuri* 65.1	8.0 ± 0.1	9.1 ± 0.1	0	0
	*S. epidermidis* 65.2	9.2 ± 0.2	9.5 ± 0.1	16.6 ± 0.2	20.4 ± 5.9
	*S. epidermidis* 4S	9.3 ± 0.2	9.5 ± 0.2	13.7 ± 2.2	44.6 ± 5.2
	*S. aureus* FRI913	9.4 ± 0.1	9.7 ± 0.2	1.4 ± 0.4	26.8 ± 2.2
	*S. aureus* CCM5757	9.4 ± 0.3	9.2 ± 0.2	0	0

**Table 11:** Reference *S. aureus* strains used in this study.

No.	Strain	Control for Genes
1	*S. aureus* FRI913	*sea*, *sec*, *sel*
2	*S. aureus* CCM5757	*seb*
3	*S. aureus* FRI1151m	*sed*
4	*S. aureus* FRI137	*seh*, *nuc*, *clf*
5	*S. epidermidis* 4S	*sec, sel*

## Data Availability

The dataset used in this study is available in the GenBank database at https://www.ncbi.nlm.nih.gov/genbank/ (last accessed: 24 February 2022). The accession numbers can be found in the text.

## Supplementary Materials

The following supporting information can be downloaded at: https://www.mdpi.com/article/10.3390/ijms23052560/s1.
